# Supplementary material for: Aspects to consider regarding breast cancer risk in trans men: A systematic review and risk management approach
Source: PLoS One. 2024 Mar 7;19(3):e0299333. doi: 10.1371/journal.pone.0299333 (PMC10919728; doi:10.1371/journal.pone.0299333)
Supplement: S1 Table — This table shows all studies excluded after full text review and states the reason behind exclusions. The inclusion criteria were as follows: (1) cases had to involve trans men, (2) breast cancer diagnosis needed to be preceded by a gender dysphoria-related intervention (either androgen therapy or a type of CCM), and/or (3) cases needed to involve invasive breast cancer or ductal carcinoma in situ. Exclusion criteria were as follows: (1) gender identity in the case subject was unclear and/or (2) a full English version of the report was unavailable. (DOCX) [file pone.0299333.s001.docx]

Suplementary table S1.

This table shows all studies excluded after full text review and states the reason behind exclusions. The inclusion criteria were as follows: (1) cases had to involve trans men, (2) breast cancer diagnosis needed to be preceded by a gender dysphoria-related intervention (either androgen therapy or a type of CCM), and/or (3) cases needed to involve invasive breast cancer or ductal carcinoma *in situ*.

Exclusion criteria were as follows: (1) gender identity in the case subject was unclear and/or (2) a full English version of the report was unavailable.

| **First author** | **Reason for exclusion** |
| --- | --- |
| Clarke et al. (2022)^1^ | Does not mention inclusion of cases, review article |
| Schultz et al. (2021)^2^ | Only benign lesions found in histopathological investigation |
| [Nishida](https://pubmed.ncbi.nlm.nih.gov/?sort=fauth&size=200&term=Nishida+M&cauthor_id=33468728) and Ishii (2021)^3^ | Full text article in japanese |
| Meggetto et al. (2019)^4^ | Does not mention inclusion of cases, review article |
| Grynberg et al. (2010)^5^ | Only benign lesions found in histopathological investigation |
| Kuroda et al. (2008)^6^ | No androgen therapy before mastectomy |
| Torous and Schnitt (2019)^7^ | Not possible to dicern if the DCIS patient were on androgen treatment |
| Hernandez et al. (2020)^8^ | No malignant lesions, however, LCIS. |
| Eckhert et al. (2020)^9^ | Breast cancer found before start of transitioning and no surgery or androgen therapy had been performed. |
| Symmers (1968)^10^ | Case reports of trans women |
| Bruce et al. (2022)^11^ | Not possible to discern if the two cases of invasive ductal carcinoma was on androgen therapy prior to diagnosis. |
| Joint et al. (2018)^12^ | Review article- including no new cases |
| Nikolić et al. (2018)^13^ | Review article-including no new cases |
| Stone et al. (2018)^14^ | Review article- including no new cases |
| Ray et al. (2020)^15^ | Review article- including no new cases |
| Baker et al. (2019)^16^ | Mentions 1 case of DCIS, this is however a duplicate of an already included case report. |
| Wolters et al. (2023)^17^ | Histopathological study, does not include any cases of invasive carcinoma or DCIS. |
| Mueller and Goreen (2008)^18^ | Review article |
| Eckhert et al. (2023)^19^ | 4 Cases are presented, however its not possible to discern if these trans men had undergone surgery or were on androgen treatment prior to diagnosis. |
| Macdonald (2019)^20^ | Includes no cases |
| van Kesteren et al. (1997)^21^ | Includes no cases |
| Pirtea et al. (2021)^22^ | Narrative review |
| Jahromi et al (2022)^23^ | Review article- including no new cases |
| Corso et al. (2023)^24^ | Review article- including no new cases |
| Traish et al.(2010)^25^ | Review article- including no new cases |
| Van Bockstal et al. (2019)^26^ | Letter to editor, no cases |
| Gurrala et al. (2023)^27^ | Review article |
| East et al. (2017)^28^ | Histopathological study, contains no cases |
| Gondusky et al. (2015)^29^ | Case from trans woman |
| Jacoby et al. (2021)^30^ | LCIS in trans men with no prior androgen therapy, incidintal find at mastectomy |
| Cortina (2022)^31^ | Includes no cases. |
| Ganly and Taylor (1995)^32^ | Case is of trans woman |
| Brown (2015)^33^ | Full text not available to authors |
| Deutsch et al. (2017)^34^ | Full text not available to authors |

1. Clarke CN, Cortina CS, Fayanju OM, et al. Breast cancer risk and screening in transgender persons: A call for inclusive care. Ann Surg Oncol. 2022; 29(4):2176-2180. doi: 10.1245/s10434-021-10217-5.
2. Schultz JJ, Naides AI, Bai D, et al. Pathological evaluation of breast specimens in transgender chest masculinization: Incidental findings and effect of prior chest binding and androgen therapy in 74 consecutive patients. Transgend Health. 2021;6(6):353-357. doi: 10.1089/trgh.2020.0108.
3. Nishida M, Ishii W. A case of breast cancer in a female-to-male transsexual during androgen therapy. Gan To Kagaku Ryoho. 2021;48(1):77-79.
4. Meggetto O, Peirson L, Yakubu M, et al. Breast cancer risk and breast screening for trans people: an integration of 3 systematic reviews. CMAJ Open. 2019;7(3):E598-E609. doi: 10.9778/cmajo.20180028.
5. Grynberg M, Fanchin R, Dubost G, et al. Histology of genital tract and breast tissue after long-term testosterone administration in a female-to-male transsexual population. Reprod Biomed Online. 2010;20(4):553-8. doi: 10.1016/j.rbmo.2009.12.021.
6. Kuroda H, Ohnisi K, Sakamoto G, et al. Clinicopathological study of breast tissue in female-to-male transsexuals. Surg Today. 2008;38(12):1067-71. doi:10.1007/s00595-007-3758-3.
7. Torous VF, Schnitt SJ. Histopathologic findings in breast surgical specimens from patients undergoing female-to-male gender reassignment surgery. Mod Pathol. 2019;32(3):346-353. doi: 10.1038/s41379-018-0117-4.
8. Hernandez A, Schwartz CJ, Warfield D, et al. Pathologic evaluation of breast tissue from transmasculine individuals undergoing gender-affirming chest masculinization. Arch Pathol Lab Med. 2020;144(7):888-893. doi: 10.5858/arpa.2019-0316-OA.
9. Eckhert E, Laniakea B, Kurian AW. A case of a trans-masculine patient receiving testosterone with a history of estrogen receptor-positive breast cancer. Breast J. 2020;26(9):1888-1889. doi: 10.1111/tbj.13829.
10. Symmers WS. Carcinoma of breast in trans-sexual individuals after surgical and hormonal interference with the primary and secondary sex characteristics. Br Med J. 1968;2(5597):83-5. doi: 10.1136/bmj.2.5597.83.
11. Bruce MK, Joseph WJ, Grunwaldt L, et al. Transgender mastectomy: incidence of high-risk pathologic findings and the need for postoperative cancer surveillance. Ann Plast Surg. 2022;88(3 Suppl 3):S148-S151. doi: 10.1097/SAP.0000000000003175.
12. Joint R, Chen ZE, Cameron S. Breast and reproductive cancers in the transgender population: a systematic review. BJOG. 2018;125(12):1505-1512. doi: 10.1111/1471-0528.15258.
13. Nikolić D, Granić M, Ivanović N, et al. Breast cancer and its impact in male transsexuals. Breast Cancer Res Treat. 2018;171(3):565-569. doi: 10.1007/s10549-018-4875-y.
14. Stone JP, Hartley RL, Temple-Oberle C. Breast cancer in transgender patients: A systematic review. Part 2: Female to Male. Eur J Surg Oncol. 2018;44(10):1463-1468. doi: 10.1016/j.ejso.2018.06.021.
15. Ray A, Fernstrum A, Mahran A, et al. Testosterone therapy and risk of breast cancer development: a systematic review. Curr Opin Urol. 2020;30(3):340-348. doi: 10.1097/MOU.0000000000000763.
16. Baker GM, Pyle ME, Tobias AM, et al. Establishing a cohort of transgender men and gender nonconforming individuals to understand the molecular impact of testosterone on breast physiology. Transgend Health. 2019;4(1):326-330. doi: 10.1089/trgh.2019.0040.
17. Wolters EA, Rabe KE, Siegel L, et al. Histopathologic features of breast tissue from transgender men and their associations with androgen therapy. Am J Clin Pathol. 2023;159(1):43-52. doi: 10.1093/ajcp/aqac123.
18. Mueller A, Gooren L. Hormone-related tumors in transsexuals receiving treatment with cross-sex hormones. Eur J Endocrinol. 2008;159(3):197-202. doi: 10.1530/EJE-08-0289.
19. Eckhert E, Lansinger O, Ritter V, et al. Breast cancer diagnosis, treatment, and outcomes of patients from sex and gender minority groups. JAMA oncology, 2023;9(4), 473–480. doi: 10.1001/jamaoncol.2022.7146.
20. Macdonald HR. Managing breast cancer risk in transgender men and women: Emerging safety data and a need for more research. Breast J. 2019;25(6):1063-1065. doi: 10.1111/tbj.13418.
21. van Kesteren PJ, Asscheman H, Megens JA, et al. Mortality and morbidity in transsexual subjects treated with cross-sex hormones. Clin Endocrinol (Oxf). 1997;47(3):337-42. doi: 10.1046/j.1365-2265.1997.2601068.x.
22. Pirtea P, Ayoubi JM, Desmedt S, et al. Ovarian, breast, and metabolic changes induced by androgen treatment in transgender men. Fertil Steril. 2021;116(4):936-942. doi: 10.1016/j.fertnstert.2021.07.1206.
23. Jahromi H, Horen S, Ho K, et al. Pathologic findings in gender-affirming mastectomy: a systematic review. Georgian Med News. 2022;(333):6-12. PMID: 36780614.
24. Corso G, Gandini S, D'Ecclesiis, et al. Risk and incidence of breast cancer in transgender individuals: a systematic review and meta-analysis. Eur J Cancer Prev. 2023;32(3), 207–214. doi:10.1097/CEJ.0000000000000784
25. Traish AM, Fetten K, Miner M, et al. Testosterone and risk of breast cancer: appraisal of existing evidence. Horm Mol Biol Clin Investig. 2010;2(1):177-90. doi: 10.1515/HMBCI.2010.024.
26. Van Bockstal MR, Monstrey SJ, van Deurzen CHM. The role of routine histopathology after chest-contouring surgery in transmen. Eur J Surg Oncol. 2019;45(3):485-486. doi: 10.1016/j.ejso.2018.10.058.
27. Gurrala RR, Kumar T, Yoo A, et al. The impact of exogenous testosterone on breast cancer risk in transmasculine individuals. Ann Plast Surg. 2023;90(1):96-105. doi: 10.1097/SAP.0000000000003321.
28. East EG, Gast KM, Kuzon WM Jr, et al. Clinicopathological findings in female-to-male gender-affirming breast surgery. Histopathology. 2017;71(6):859-865. doi: 10.1111/his.13299.
29. Gondusky CJ, Kim MJ, Kalantari BN, et al. Examining the role of screening mammography in men at moderate risk for breast cancer: two illustrative cases. Breast J. 2015;21(3):316-7. doi: 10.1111/tbj.12411.
30. Jacoby A, Rifkin W, Zhao LC, et al. Incidence of cancer and premalignant lesions in surgical specimens of transgender patients. Plast Reconstr Surg. 2021;147(1):194-198. doi: 10.1097/PRS.0000000000007452.
31. Cortina CS. Inclusion and reporting of transgender and nonbinary persons in clinical trials and tumor registries-the time is now. JAMA Oncol. 2022;8(8):1097-1098. doi: 10.1001/jamaoncol.2022.1638.
32. Ganly I, Taylor EW. Breast cancer in a trans-sexual man receiving hormone replacement therapy. Br J Surg. 1995;82(3):341. doi: 10.1002/bjs.1800820319.
33. Brown GR. Breast cancer in transgender veterans: A ten-case series. LGBT Health. 2015;2(1):77-80. doi: 10.1089/lgbt.2014.0123.
34. Deutsch MB, Radix A, Wesp L. Breast cancer screening, management, and a review of case study literature in transgender populations. Semin Reprod Med. 2017;35(5):434-441. doi: 10.1055/s-0037-1606103.
