# Supplementary material for: Aspects to consider regarding breast cancer risk in trans men: A systematic review and risk management approach
Source: PLoS One. 2024 Mar 7;19(3):e0299333. doi: 10.1371/journal.pone.0299333 (PMC10919728; doi:10.1371/journal.pone.0299333)
Supplement: S2 Table — This table describes the search process as conducted on PubMed. The search was constructed through 7 steps. The combined and final search were as follows: ("breast cancer"[Text Word] OR "breast malignancy"[Text Word] OR "breast Neoplasms"[Text Word] OR "Breast carcinoma"[Text Word] OR ("breast"[Text Word] AND "carcinoma"[Text Word]) OR ("breast Neoplasms"[MeSH Terms] OR "carcinoma, ductal, breast"[MeSH Terms] OR "breast carcinoma in situ"[MeSH Terms])) AND ("transgender"[Text Word] OR "transgender male*"[Text Word] OR "female-to-male"[Text Word] OR "female-to-male"[Text Word] OR "transexual*"[Text Word] OR "trans man"[Text Word] OR "trans men"[Text Word] OR ("transgender persons*"[MeSH Terms] OR "transsexualism*"[MeSH Terms] OR "gender identity"[MeSH Terms])). The search was conducted on the 14th of march 2023. It resulted in 374 hits. (DOCX) [file pone.0299333.s002.docx]

Supplementary table S2.

This table describes the search process as conducted on PubMed. The search was constructed through 7 steps. The combined and final search were as follows: ("breast cancer"[Text Word] OR "breast malignancy"[Text Word] OR "breast Neoplasms"[Text Word] OR "Breast carcinoma"[Text Word] OR ("breast"[Text Word] AND "carcinoma"[Text Word]) OR ("breast Neoplasms"[MeSH Terms] OR "carcinoma, ductal, breast"[MeSH Terms] OR "breast carcinoma in situ"[MeSH Terms])) AND ("transgender"[Text Word] OR "transgender male*"[Text Word] OR "female-to-male"[Text Word] OR "female-to-male"[Text Word] OR "transexual*"[Text Word] OR "trans man"[Text Word] OR "trans men"[Text Word] OR ("transgender persons*"[MeSH Terms] OR "transsexualism*"[MeSH Terms] OR "gender identity"[MeSH Terms])). The search was conducted on the 14^th^ of march 2023. It resulted in 374 hits.

| **Search** | **Actions** | **Details** | **Query** | **Results** | **Time** |
| --- | --- | --- | --- | --- | --- |
| #7 |  |  | Search: **#5 AND #6** | [374](https://pubmed.ncbi.nlm.nih.gov/?term=%235+AND+%236&sort=&size=200) | 06:22:27 |
| #6 |  |  | Search: **#3 OR #4** | [38,970](https://pubmed.ncbi.nlm.nih.gov/?term=%233+OR+%234&sort=&size=200) | 06:22:09 |
| #5 |  |  | Search: **#1 OR #2** | [448,590](https://pubmed.ncbi.nlm.nih.gov/?term=%231+OR+%232&sort=&size=200) | 06:21:52 |
| #4 |  |  | Search: **"transgender persons*"[mh] OR transsexualism*[mh] OR "gender identity"[mh]** | [30,661](https://pubmed.ncbi.nlm.nih.gov/?term=%22transgender+persons%2A%22%5Bmh%5D+OR+transsexualism%2A%5Bmh%5D+OR+%22gender+identity%22%5Bmh%5D&sort=&size=200) | 06:21:33 |
| #3 |  |  | Search: **transgender[tw] OR "transgender male*"[tw] OR "female-to-male"[tw] OR "female to male"[tw] OR Transexual*[tw] OR "trans man"[tw] OR "trans men"[tw]** | [16,133](https://pubmed.ncbi.nlm.nih.gov/?term=transgender%5Btw%5D+OR+%22transgender+male%2A%22%5Btw%5D+OR+%22female-to-male%22%5Btw%5D+OR+%22female+to+male%22%5Btw%5D+OR+Transexual%2A%5Btw%5D+OR+%22trans+man%22%5Btw%5D+OR+%22trans+men%22%5Btw%5D&sort=&size=200) | 06:21:17 |
| #2 |  |  | Search: **"breast neoplasms"[mh] OR "carcinoma, ductal, breast"[mh] OR "breast carcinoma in situ"[mh]** | [338,498](https://pubmed.ncbi.nlm.nih.gov/?term=%22breast+neoplasms%22%5Bmh%5D+OR+%22carcinoma%2C+ductal%2C+breast%22%5Bmh%5D+OR+%22breast+carcinoma+in+situ%22%5Bmh%5D&sort=&size=200) | 06:21:00 |
| #1 |  |  | Search: **"breast cancer"[tw] OR "breast malignancy"[tw] OR "breast Neoplasms"[tw] OR "Breast carcinoma"[tw] OR (breast[tw] AND carcinoma[tw])** | [447,572](https://pubmed.ncbi.nlm.nih.gov/?term=%22breast+cancer%22%5Btw%5D+OR+%22breast+malignancy%22%5Btw%5D+OR+%22breast+Neoplasms%22%5Btw%5D+OR+%22Breast+carcinoma%22%5Btw%5D+OR+%28breast%5Btw%5D+AND+carcinoma%5Btw%5D%29&sort=&size=200) | 06:20:41 |
